# Supplementary figures and images for: Spatial gene expression maps of the intestinal lymphoid follicle and associated epithelium identify zonated expression programs
Source: PLoS Biol. 2021 Oct 11;19(10):e3001214. doi: 10.1371/journal.pbio.3001214 (PMC8530339; doi:10.1371/journal.pbio.3001214)

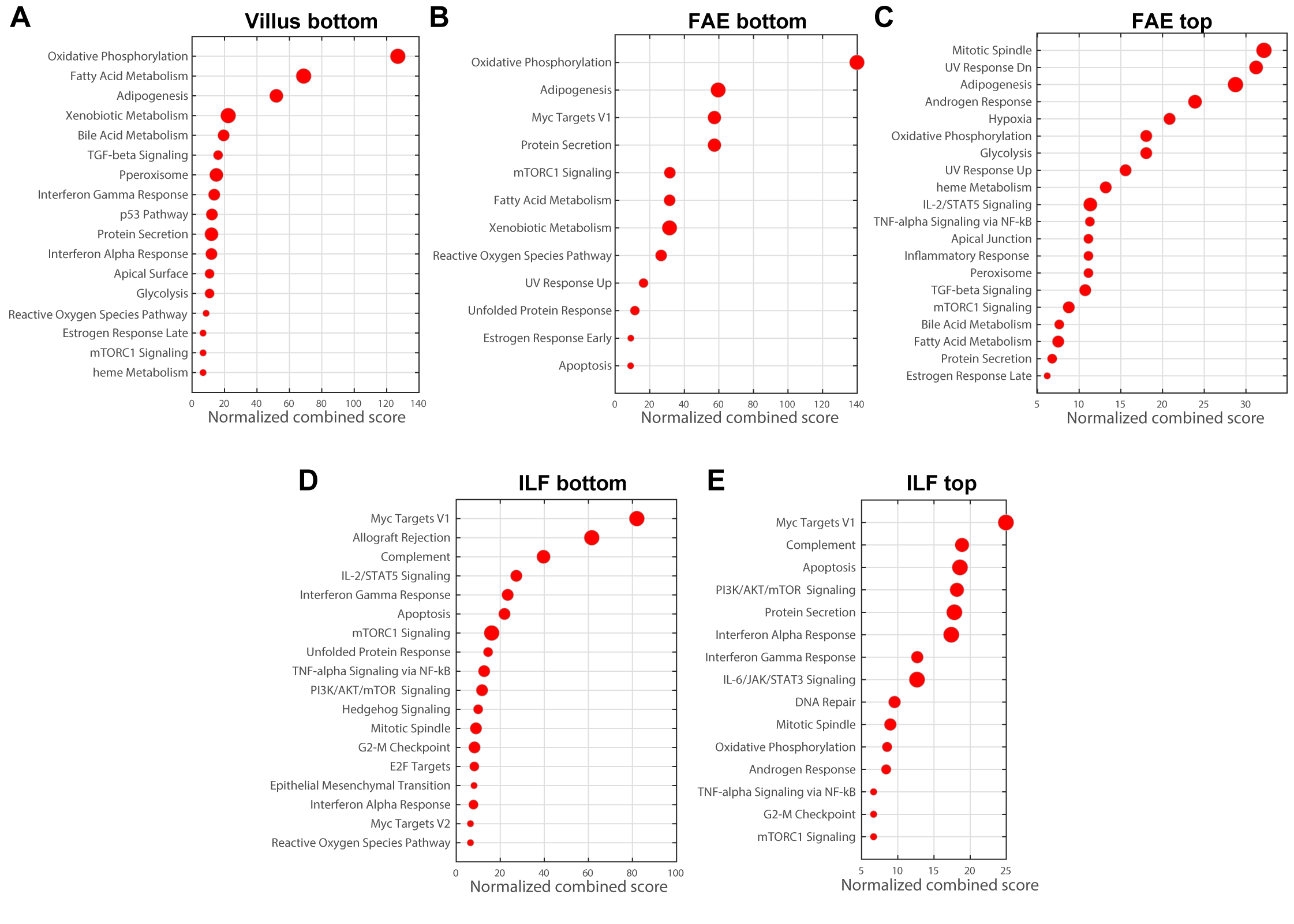

Supplement: S1 Fig — Related to Fig 1. (A-E) Dot plots generated by Enrichr (see Methods) showing normalized combined score (with q-value less than 0.1) of up-regulated gene sets for VB, FAEB, FAET, ILFB, and ILFT. The size of each red dot is in accordance with the number of genes in each set. The data used to generate this figure can be found in Supporting information S3 Data. FAEB, FAE bottom; FAET, FAE top; ILFB, ILF bottom; ILFT, ILF top; VB, villus bottom. (TIF) [file pbio.3001214.s001.tif]

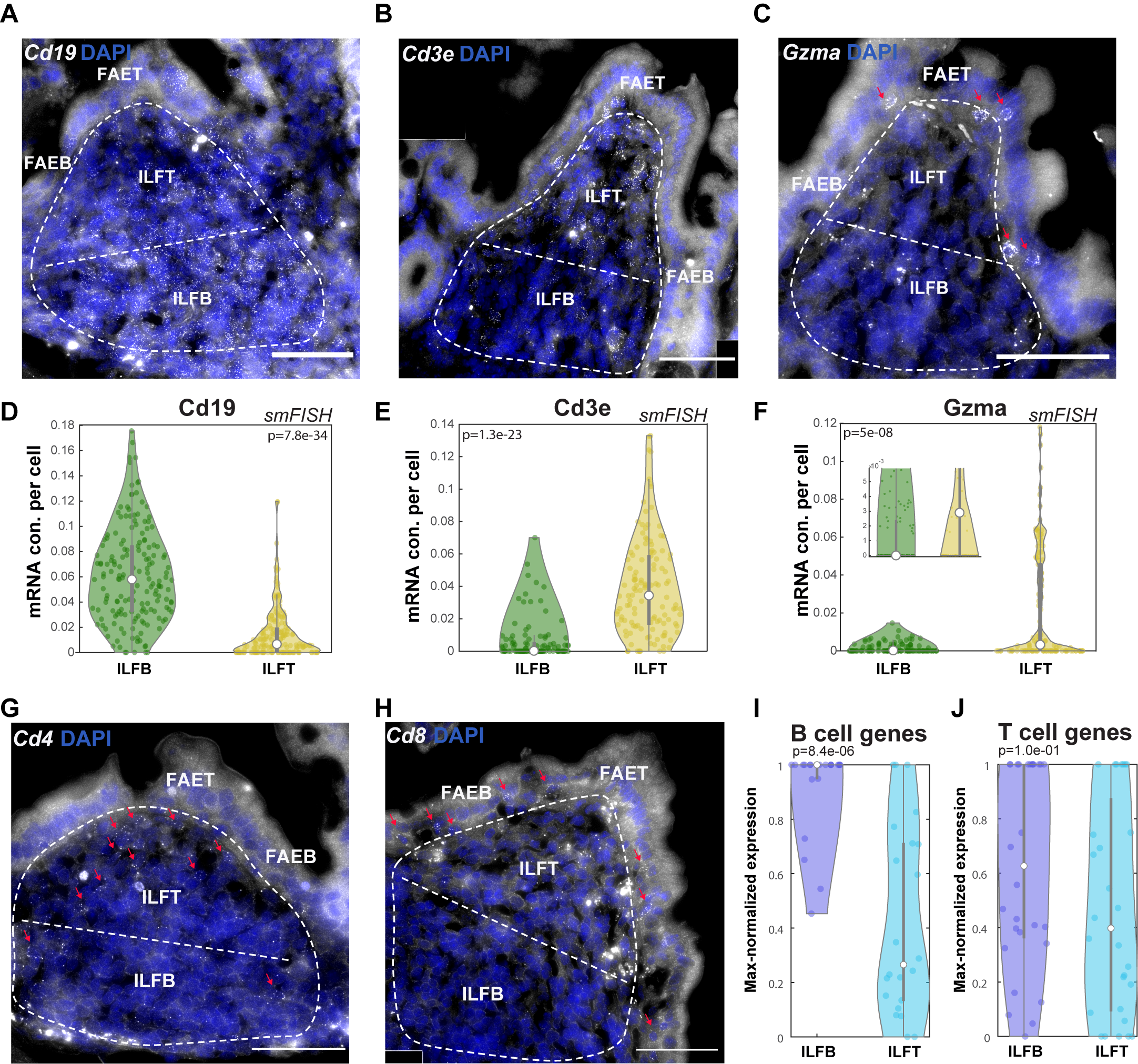

Supplement: S2 Fig — Related to Fig 1. (A-C, G-H) smFISH validations showing increased expression of Cd19 (A) at the ILFB and Cd3e (B), Gzma (C), Cd4 (G), Cd8 (H) at the ILFT. White dashed lines delimit segment ILF areas, and a border line in the middle separates ILFT and ILFB. Red arrows highlight cells with elevated expression of the respective genes. DAPI staining for cell nucleus in blue. Scale bar: 50 μm. (D-F) Violin plots of dot quantifications of smFISH signals of Cd19, Cd3e, and Gzma, showing the concentration (con.) of dots (mRNA molecules) per cell area (3–5 individual ILF per mouse for 4 mice). Blowup in (F) highlights the majority of the cells with lower expression levels, demonstrating the increase in median Gzma levels at the ILFT. (I) Violin plots showing max-normalization of B and T cell signature gene expression (Methods). B cell markers are significantly zonated to the ILFB, whereas T cell markers do not exhibit significant bias to the ILFB. White dots are median values; gray boxes delineate the 25–75 percentiles, p-values computed using Kruskal–Wallis tests. The data used to generate this figure can be found in Supporting information S2, S5, and S6 Data. FAEB, FAE bottom; FAET, FAE top; ILF, isolated lymphoid follicle; ILFB, ILF bottom; ILFT, ILF top; smFISH, single-molecule fluorescence in situ hybridization. (TIF) [file pbio.3001214.s002.tif]

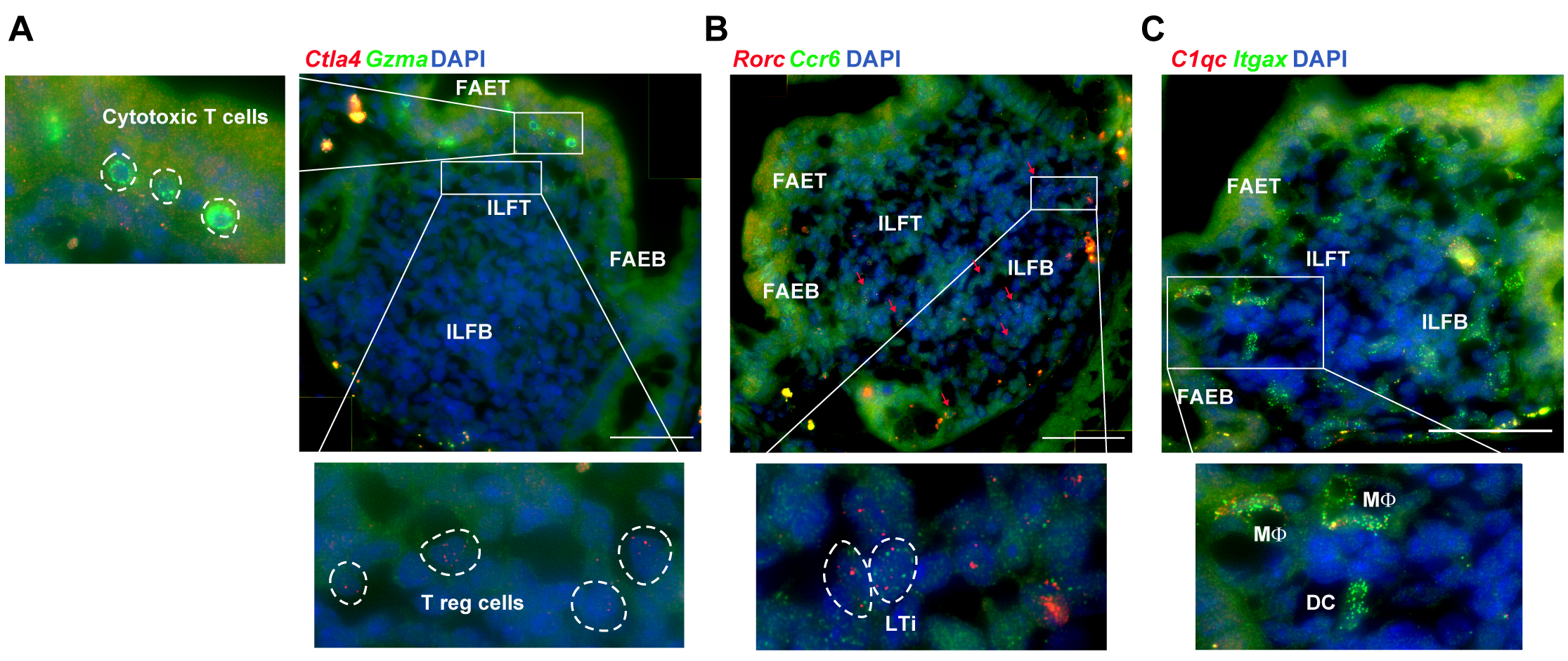

Supplement: S3 Fig — Related to Fig 1. (A-C) smFISH images and blowups showing Gzma expressing cytotoxic T cells at the ILFT, infiltrating to the FAET (A), as well as Ctla4+ Tregs (A) and Rorc+Ccr6+ LTi cells (B) that are scattered throughout the ILFs. Red arrows highlight representative cells. (C) Dendritic cells (Itgax+C1qc− cells) and macrophages (Mϕ, Itgax+C1qc+ cells) are radially zonated toward the periphery of the ILF. Scale bar- 50 μm. DC, dendritic cell; FAEB, FAE bottom; FAET, FAE top; ILF, isolated lymphoid follicle; ILFB, ILF bottom; ILFT, ILF top; LTi, lymphoid tissue–induced; smFISH, single-molecule fluorescence in situ hybridization; Treg, regulatory T cell. (TIF) [file pbio.3001214.s003.tif]

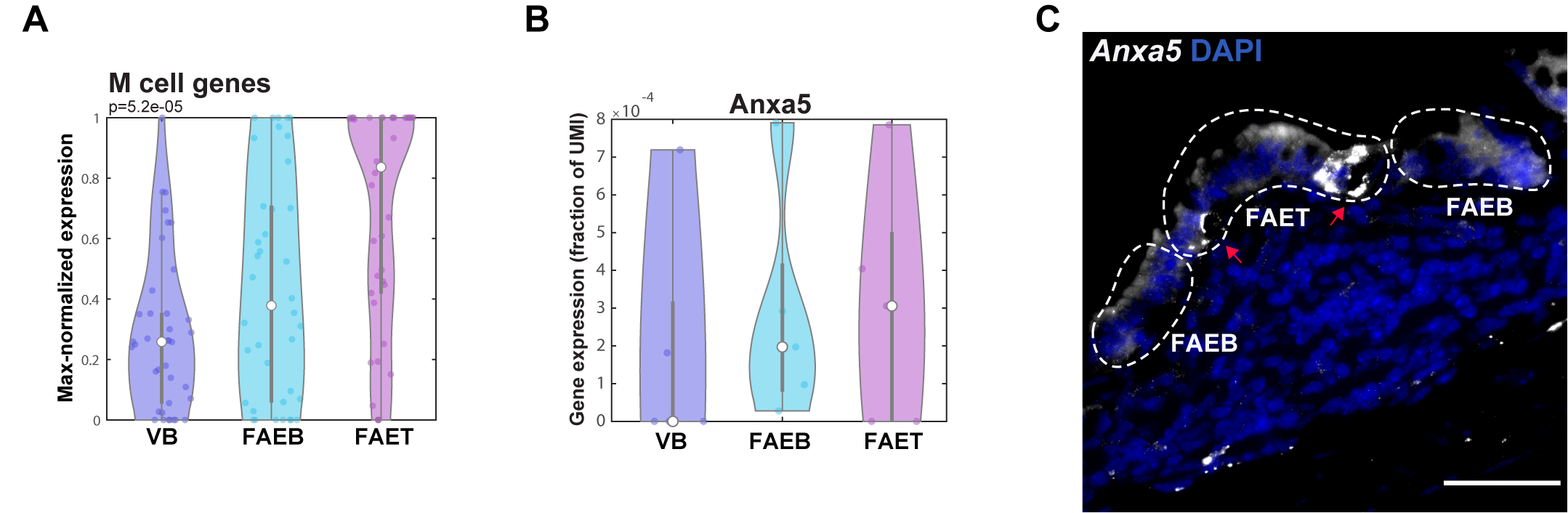

Supplement: S4 Fig — Related to Fig 1 (A) Violin plots for the max-normalized expression of M cell signature genes (Methods) showing an up-regulation expression of M cell genes in FAET compared to FAEB and VB. (B) Violin plot showing up-regulation of Anxa5 expression in FAET compared to FAEB and VB. Five FAE zones from 2 mice. In A, B, white dots are median values; gray boxes delineate the 25–75 percentiles, p-values computed using Kruskal–Wallis tests. P > 0.05 for Anxa5 (B). (C) A smFISH image showing Anxa5 expression in the FAET. White dashed lines delimit FAET and FAEB areas, and red arrows mark cells with higher expression levels of Anxa5. Scale bar: 50 μm. The data used to generate this figure can be found in Supporting information S2 and S6 Data. FAE, follicle-associated epithelium; FAEB, FAE bottom; FAET, FAE top; smFISH, single-molecule fluorescence in situ hybridization; UMI, unique molecular identifier; VB, villus bottom. (TIF) [file pbio.3001214.s004.tif]

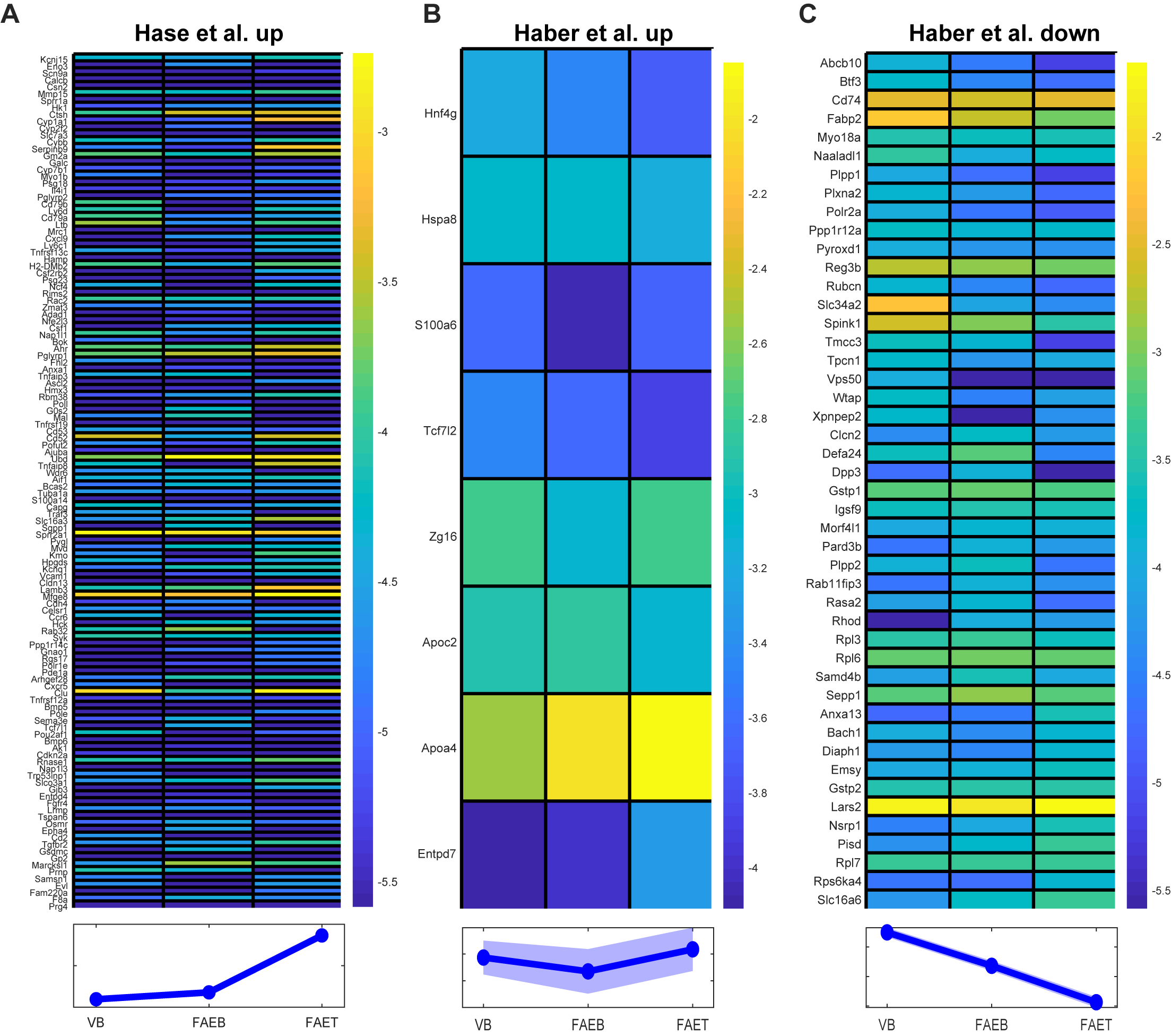

Supplement: S5 Fig — Related to Fig 1. (A) Log10 of the average expression in each of the epithelial zones for RefSeq genes previously shown to be differentially expressed in FAE of Peyer’s patches [9]. (B, C) Log10 of the average expression of genes that are differentially expressed between FAE enterocytes and villus enterocytes in the jejunum ([8]; Methods). Bottom plots show mean of log10 expression of the gene sets; patches are standard errors of the means. The data used to generate this figure can be found in S1 Data. FAE, follicle-associated epithelium; FAEB, FAE bottom; FAET, FAE top; VB, villus bottom. (TIF) [file pbio.3001214.s005.tif]

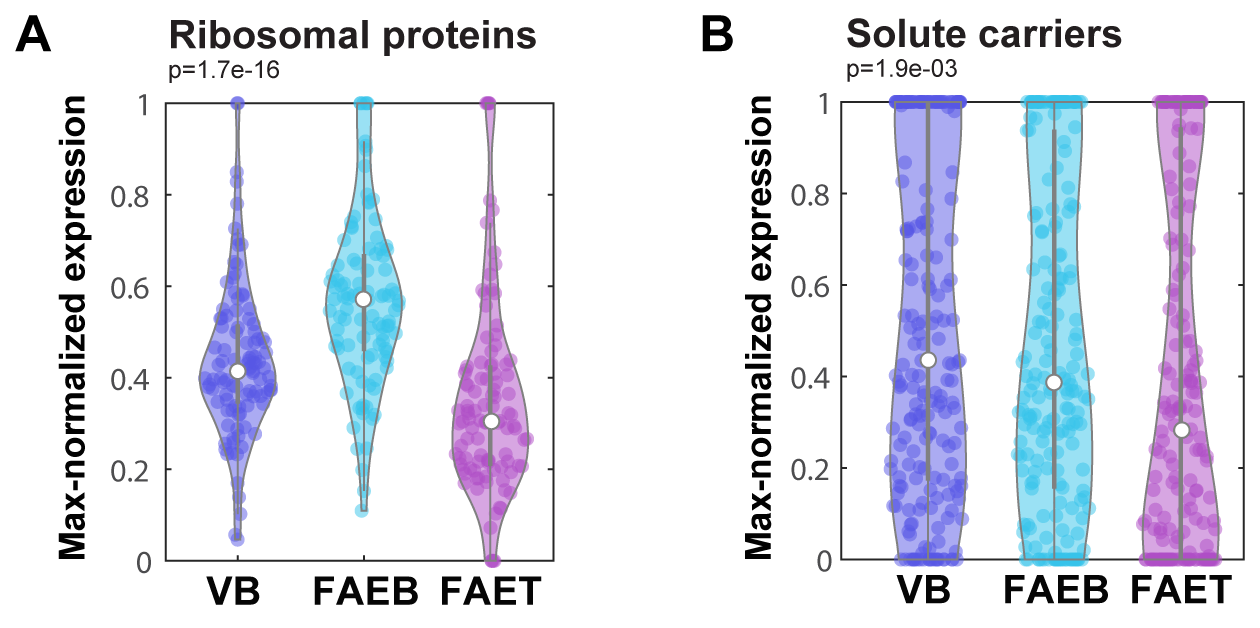

Supplement: S6 Fig — Related to Fig 2. (A, B) Violin plots showing down-regulated max-normalized expression of ribosomal proteins (A) and solute carriers (B) in FAET compared to FAEB and VB. P values of Kruskal–Wallis tests are presented. White dots represent median values. The data used to generate this figure can be found in Supporting information S2 and S6 Data. FAE, follicle-associated epithelium; FAEB, FAE bottom; FAET, FAE top; VB, villus bottom. (TIF) [file pbio.3001214.s006.tif]
