# Supplementary material for: Spatial gene expression maps of the intestinal lymphoid follicle and associated epithelium identify zonated expression programs
Source: PLoS Biol. 2021 Oct 11;19(10):e3001214. doi: 10.1371/journal.pbio.3001214 (PMC8530339; doi:10.1371/journal.pbio.3001214)
Supplement: Attachment — Submitted filename: Itzkovitz_response_to_reviewer_comments.pdf [file pbio.3001214.s013.pdf]

Dear Ines,

We thank you and the reviewers for your excellent and constructive comments. We have now addressed all of the points raised in the revised manuscript with new experiments and analyses. Specifically, we have now performed computational deconvolution to obtain an unbiased assessment of cell composition in each zone, performed all smFISH experiments on two additional mice to obtain four mice overall and explored the in-situ spatial gene expression patterns of additional immune cell types in the ILFs. We believe these new results and analyses significantly strengthen our work.

Below we provide our detailed responses; modified text in the paper is underlined.

As you will see, the reviewers find the results novel and interesting, but they also raise several issues that will need to be addressed. Reviewer 1 would like to see experiments addressing the function of the Lepr+ telocyte population, and changes in the structure in germ free animals or disease models or humans. However, after discussing these experiments with the academic editor, we do not think they are necessary for a Short Report. Nevertheless, we do agree with this reviewer that you should do more analysis of the immune cell types present as we consider this quite key for the spatial organisation of the structure. In addition, we would expect you to address the following points in a revision:

Reviewer 1:

1. You should compare your data to the studies analyzing the transcriptome of FAE in Peyer's Patches (manuscript references 8-10).

We have now included a new supplementary figure that compares our results to previous transcriptomics studies of Peyer's patches:

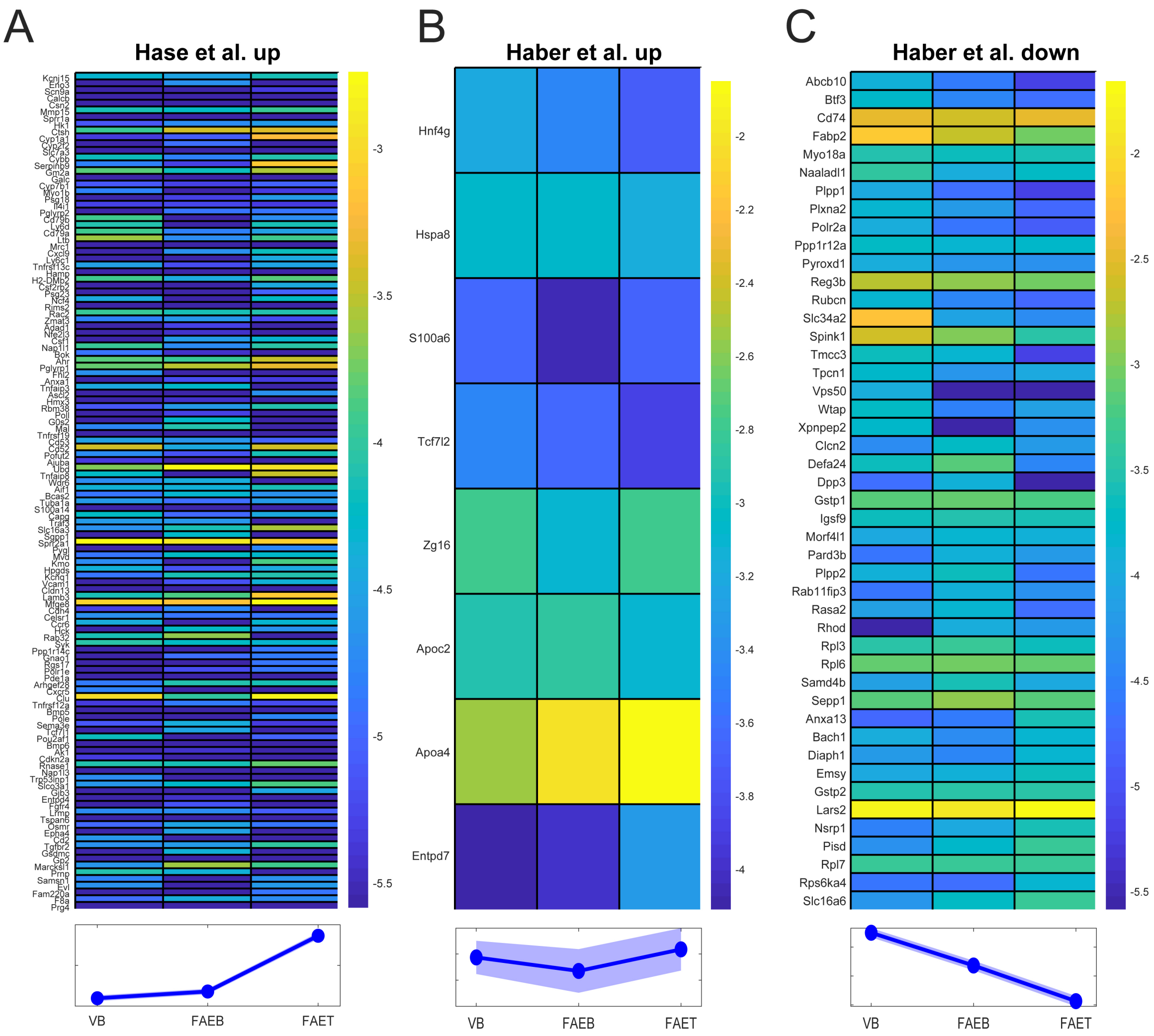

S5 Fig. Representation of genes previously shown to be differentially expressed in FAE in Peyer's Patches. Related to Fig 1. (**A**) Log10 of the average expression in each of the epithelial zones for RefSeq genes previously shown to be differentially expressed in FAE of Peyer's Patches [9]. (B-C) log10 of the average expression of genes that are differentially expressed between FAE enterocytes and villus enterocytes in the jejunum ([8], Methods). Bottom plots show mean of log10 expression of the gene sets, patches are standard errors of the means. The data used to generate this figure can be found in supporting information S1.

This analysis is described on page 3:

"The FAE top showed higher levels of genes previously shown to be elevated in FAE of Peyer's patches ([9], S5A Fig) and lower levels of genes shown to be down-regulated in FAE of Peyer's Patches ([8], S5B-C Fig, Methods). Our deconvolution analysis indicated

that the FAE top segment was enriched in enterocytes with expression programs typical of the villus top (Fig 1E). We next turned to examine the zonated properties of the non-M cell epithelial cells, constituting 90% of this tissue compartment."

2. Which T cell types are present?

We have addressed this question with new smFISH analyses of specific immune and T cell markers, as well as with computational deconvolution, as suggested by reviewer 2.

For the computational deconvolution we have parsed a single cell dataset of the small intestine and used the extracted cell-specific transcriptomic signatures as input to the CIBERSORTx deconvolution software (new Figure 1E and new Supplementary Dataset, S1 data). This provided important insights into the spatial distribution of distinct immune subsets in the ILFs:

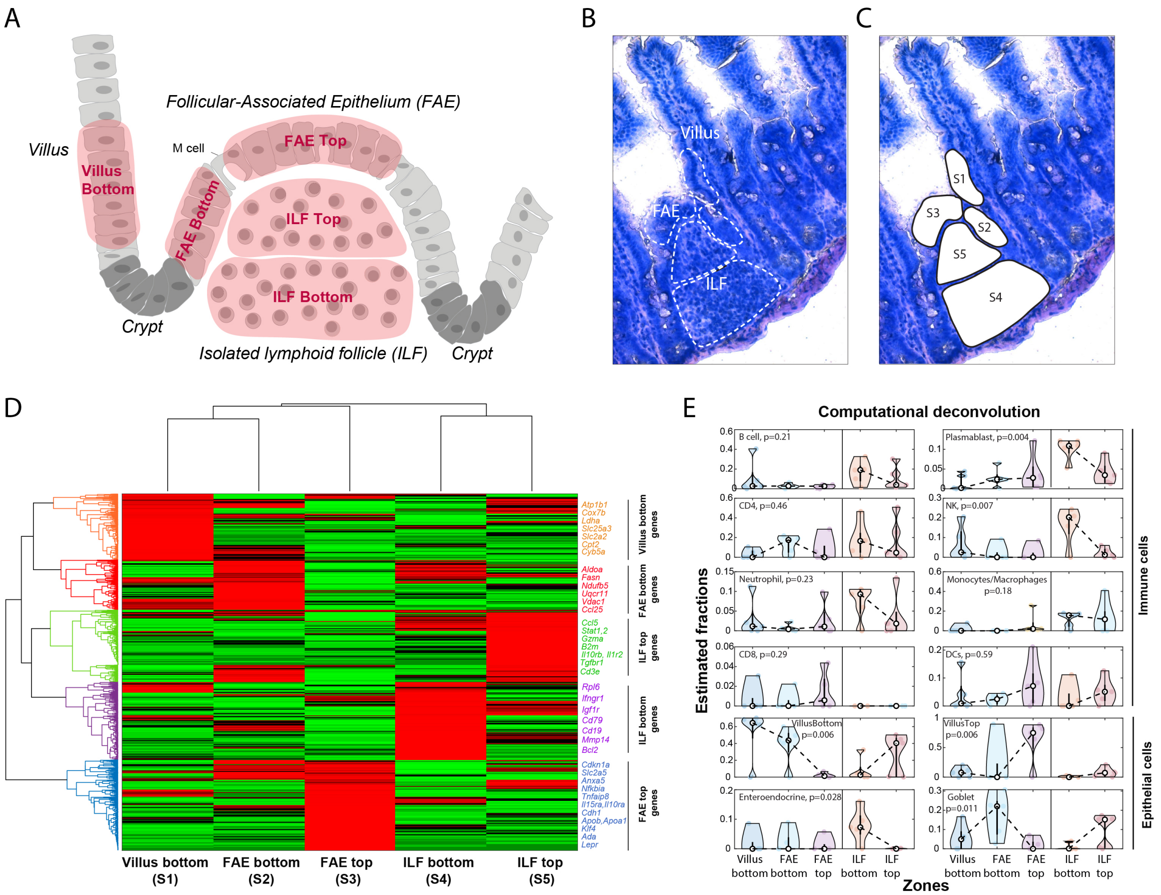

Fig 1. **LCM RNA-seq of follicle-associated epithelium (FAE) and isolated lymphoid follicle (ILF)**. (**A**) An illustration of small intestinal region including FAE, ILF, adjacent villus and crypts, showing the five dissected segments in red: Villus bottom (S1), FAE bottom (S2), FAE top (S3), ILF bottom (S4) and ILF top (S5). (**B-C**) A bright field microscopy image (20x magnification) of FAE, ILF and the adjacent villus before and after laser dissection of the five segments (S1-S5). (**D**) Clustergram of LCM RNA-seq data showing gene mean expression Z-score of the five segments (S1-S5). Selected genes with high expression are shown on the right side of the clustergram for each cluster, colored according to the cluster color. (**E**) Estimated fractions of distinct cell types, based on computational deconvolution of the LCMseq. White dots are medians, black boxes delineate the 25-75 percentiles. The data used to generate this figure can be found in supporting information S1 and S2 data. P-values computed using Kruskal Wallis tests.

This new analysis is described on page 3:

"To assess the cellular composition of each of segment we performed computational deconvolution using the CIBERSORTx tool [17], that included epithelial and immune single cell-based signatures ([8,11], Fig 1E, S1 data). Among the immune cells, Plasmablasts, B cells, neutrophils and NK cells were more abundant at the ILF bottom, whereas CD8 T cells and dendritic cells were more highly represented (yet not statistically significantly) at the ILF top or the FAE top."

We have also now specifically explored the expression of key T-cell genes – Gzma, Cd4, Cd8a and Ctla4. These genes exhibited interesting zonated trends and are shown in the new Figs S2, S3:

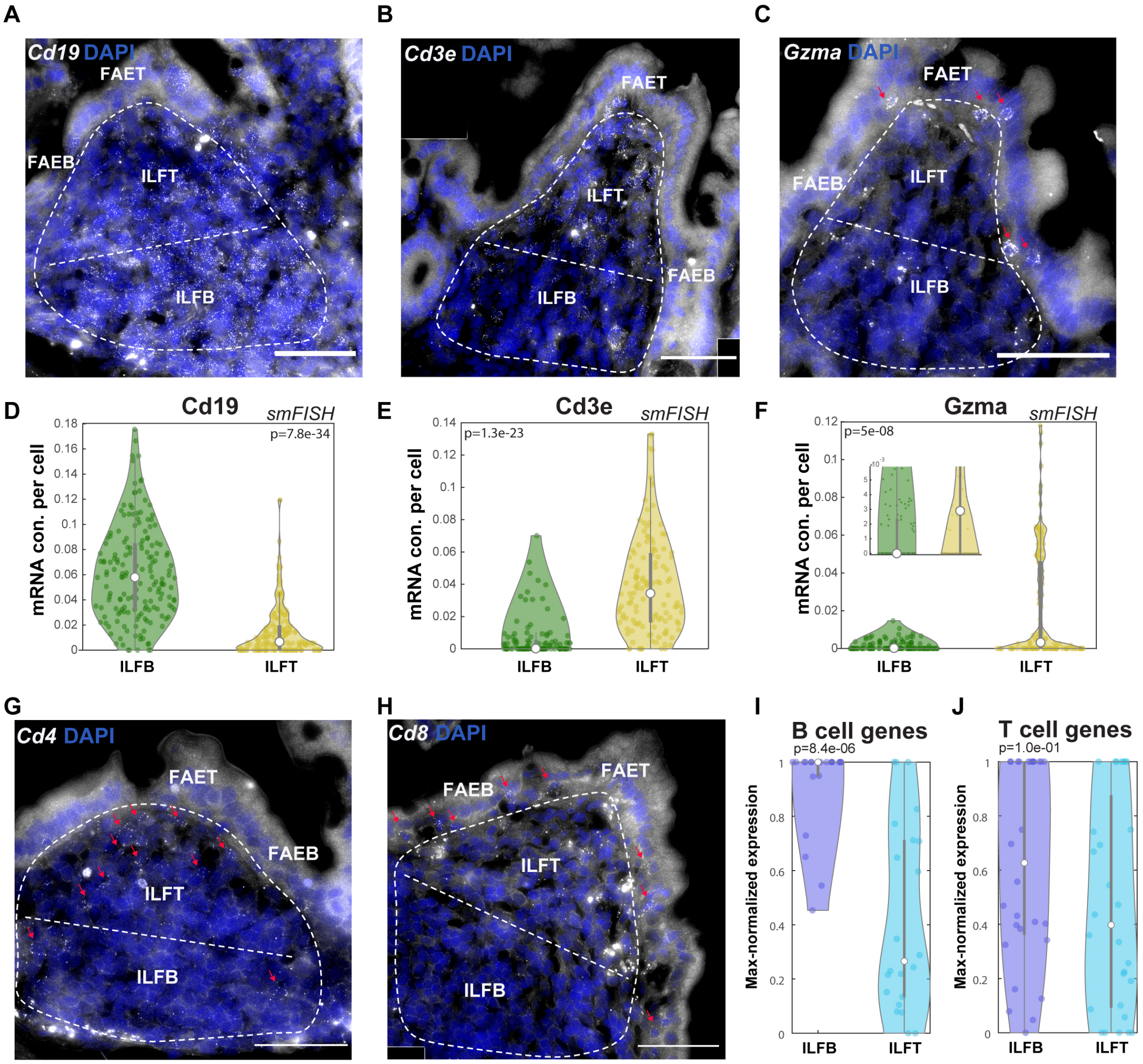

S2 Fig. Zonation of B and T cells in ILF. Related to Fig 1. (**A-C, G-H**) smFISH validations showing increased expression of *Cd19* (**A**) at the ILF bottom and *Cd3e* (**B**), *Gzma* (**C**), *Cd4* (**G**), *Cd8* (**H**) at the ILF top. White dashed lines delimit segment ILF areas and a border line in the middle separates ILFT and ILFB. Red arrows highlight cells with elevated expression of the respective genes. DAPI staining for cell nucleus in blue. Scale bar- 50 µm. (**D-F**) Violin plots of dot quantifications of smFISH signals of *Cd19*, *Cd3e* and *Gzma*, showing the concentration (con.) of dots (mRNA molecules) per cell area (3-5 individual ILF per mouse for 4 mice). Blow up in **(F)** highlights the majority of the cells with lower expression levels, demonstrating the increase in median *Gzma* levels at the ILFT. (**I**) Violin plots showing max-normalization of B and T cell signature gene expression (Methods). B cell markers are significantly zonated to the ILFB, whereas T cell markers do not exhibit significant bias to the ILFB. White dots are median values, gray boxes delineate the 25-75 percentiles, p-values computed using Kruskal Wallis tests. The data used to generate this figure can be found in supporting information S2, S5 and S6 data.

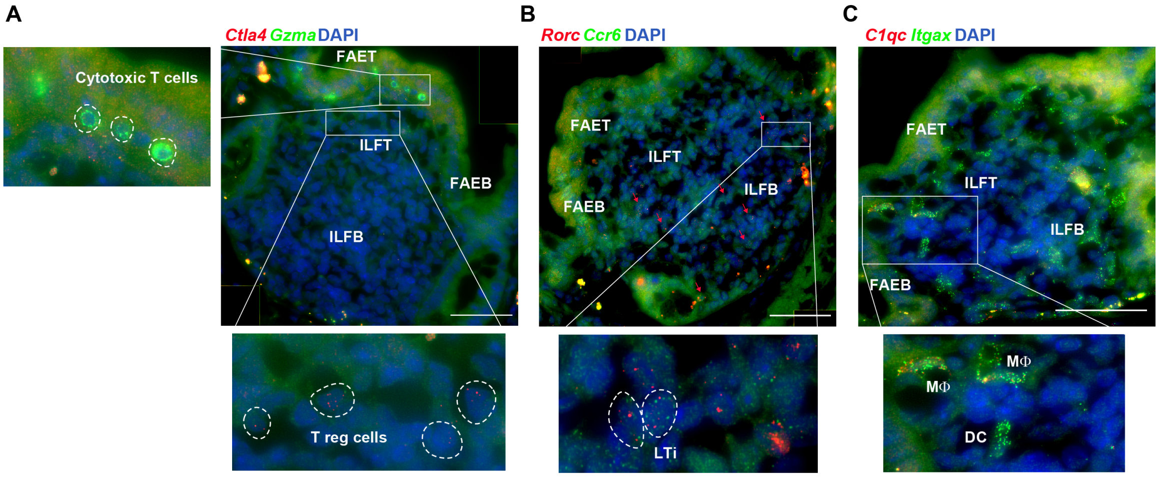

S3 Fig. Immune cell subsets in ILFs. Related to Fig 1. (**A-C**) smFISH images and blow ups showing *Gzma* expressing cytotoxic T cells at the ILF top, infiltrating to the FAET (**A**), as well as *Ctla4*+ regulatory T cells (Treg, **A**) and *Rorc*+*Ccr6*+ LTi cells (**B**) that are scattered throughout the ILFs. Red arrows highlight representative cells. (**C**) Dendritic

cells (Itgax+C1qc- cells) and macrophages (Mϕ, Itgax+C1qc+ cells) are radially zonated towards the periphery of the ILF. Scale bar- 50 µm.

These are discussed on page 3:

"Consistently, we identified higher expression of different B-cell markers, such as *Cd19,* at the bottom of the ILF (S2A,D,I Fig). In contrast, T cell markers did not show a spatial bias towards a specific zone in the ILF (S2J Fig). Rather, using smFISH we found that *Cd3e, Cd4 and Cd8,* classic T cell markers, as well as *Gzma*, a marker of cytotoxic CD8+ T cells were highly abundant at the ILF top. (S2B,C,E-H Fig). Consistently, CD8+ and Gzma+ cells were abundantly intercalated across the FAE top (S3A Fig), whereas CD4+ cells (S2G Fig) as well as the Treg marker Ctla4 were zonated towards the ILF top (S3A Fig). The spatial pattern of lymphocytes in the ILF resembles the architecture previously observed in Peyer's patches, which exhibit a core of B cells and a mantle of T cells [18]."

3. Where are the dendritic cells and the LTis?

We have now performed new smFISH experiments to identify the rare LTi cells, using co-smFISH of the markers Rorc and Ccr6. Indeed, we identify these cells as rare and scattered, without specific apparent zonation patterns. We have also now performed new smFISH experiments with the probe libraries for the genes Itgax and C1qc. The combined visualization of both of these genes enables identification of dendritic cells (Itgax+C1qc- cells) and macrophages (Itgax+C1qc+ cells). Interestingly, we found that both dendritic cells and macrophages have a radial zonation, enriched towards the periphery of the ILFs. These results are shown in the new S3B,C Fig above and described on page 3:

"Using smFISH, we further identified scatter localization of Rorc+Ccr6+ Lymphoid Tissue induced cells (LTi cells [19], S3B Fig), as well as radial zonation of dendritic cells (identified as Itgax+C1qc- cells) and macrophages (Itgax+C1qc+ cells) [20] towards the periphery of the ILF (S3C Fig)"

Reviewer 2:

1. The point regarding the low number of samples and statistical analyses should be

addressed, particularly for the smFISH which seems to be only from two mice, but is subjected to statistical analysis. Are these statistics valid? Standard convention is to have three biological replicates prior to statistical treatment of the data.

We thank you and the reviewer for this important comment. We have now repeated all of our smFISH experiments over two additional mice so that we are now presenting results based on quantification of four mice. All revised figures have been updated accordingly. All original conclusions have remained unchanged. We have updated all figure captions and Methods with this new number of mice, and present all of the smFISH quantification of single cells in the new Supplementary Dataset (S5 data).

2. Please avoid jargon in the manuscript to make it accessible to a broad audience.

We have now modified the text to improve clarity, specifically avoiding jargon related to the transcriptomics part.

Minor:
In Supplementary Figure 3E, CD3a is increased at ILFT (not at ILFB) - the figure legend seems incorrect while the main text is correct.
We have now corrected this.
In addition, on Supplementary Fig. 3E the medians for Gmza for ILFT and ILFB seem very similar according to the violin plot.
We have now added a blowup of the area with lower expression values, demonstrating the clear increase in median Gzma expression at the ILFT.

Reviewers' comments

Rev. 1:
In their manuscript Cohen et al, made a short and concise description of the expression

profiles of isolated lymphoid follicles and their associated epithelium. The authors use a combination of laser capture micro dissection, RNAsequencing and smFISH to identify areas with unique expression profiles. The authors also describe the identification of Lepr+ sub-epithelial telocytes.

The study shows that according to their transcriptome profile ILFs could be divided into different compartments, which is a novel and interesting information for mucosal immunologists and epithelial biologists, but provides limited information on the biological relevance of such finding. There is no further insight into the function of ILF, FAE or the newly described Lepr+ sub-epithelial telocytes.

The authors should compare their data to the studies analysing the transcritptome of FAE in Peyer's Patches (manuscript references 8-10).

We thanks the reviewer for this suggestion, we have now included a new supplementary figure that compares our results to previous transcriptomics studies performed on Peyer's patches:

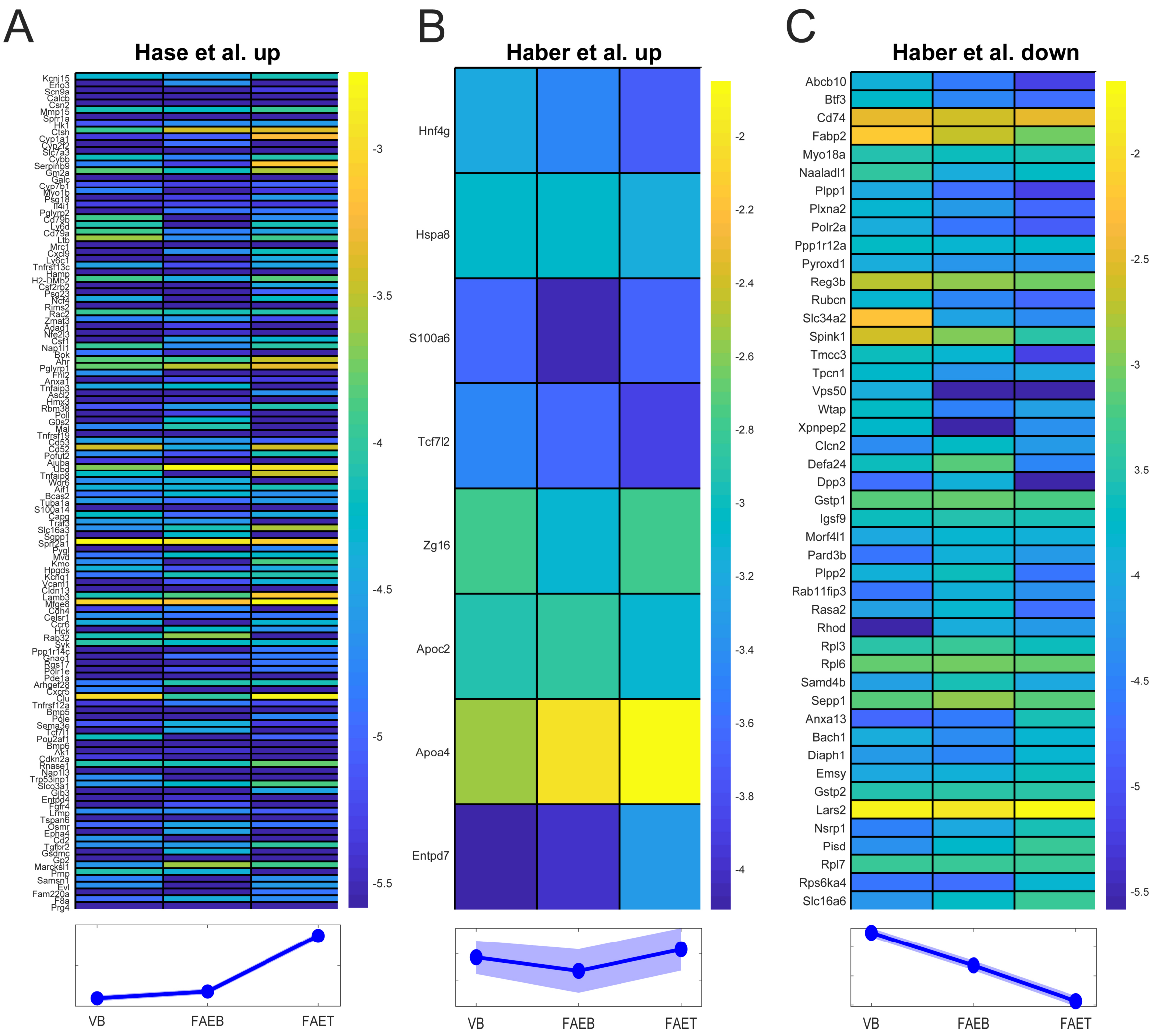

S5 Fig. Representation of genes previously shown to be differentially expressed in FAE in Peyer's Patches. Related to Fig 1. (**A**) Log10 of the average expression in each of the epithelial zones for RefSeq genes previously shown to be differentially expressed in FAE of Peyer's Patches [9]. (B-C) log10 of the average expression of genes that are differentially expressed between FAE enterocytes and villus enterocytes in the jejunum ([8], Methods). Bottom plots show mean of log10 expression of the gene sets, patches are standard errors of the means. The data used to generate this figure can be found in supporting information S1.

This analysis is described on page 3:

"The FAE top showed higher levels of genes previously shown to be elevated in FAE of Peyer's patches ([9], S5A Fig) and lower levels of genes shown to be down-regulated in

FAE of Peyer's Patches ([8], S5B-C Fig, Methods). Our deconvolution analysis indicated that the FAE top segment was enriched in enterocytes with expression programs typical of the villus top (Fig 1E). We next turned to examine the zonated properties of the non-M cell epithelial cells, constituting 90% of this tissue compartment."

And in the Methods section on page 15:

"The list of epithelial genes previously shown to be up-regulated in FAE of Peyer's patches using microarray measurements ([9], S5A Fig) was generously provided by Hiroshi Saito and Koji Hase. Epithelial genes up- or down-regulated in FAE of Peyer's patches based on Haber et al. ([8], S5B-C Fig) were extracted as follows – FAE cells were extracted from the FAE scRNAseq dataset and defined as cells annotated as "Enteroproximal" with UMI sum-normalized expression of the villus top gene Apoa1 above 10^{-3}. Villus cells were extracted from the Regional scRNAseq dataset and defined as jejunum cells with UMI sum-normalized expression above 10^{-3}. The two datasets were normalized over the set of intersecting genes, after removal of epithelial secretory cell genes, defined as genes with expression in epithelial secretory cells that is larger than 10^{-5} and larger than 2-fold the expression in enterocytes. To this end, epithelial secretory cell gene expression was defined as the maximal mean expression among Paneth cells, tuft cells goblet cells and enteroendocrine cells, extracted from Haber et al. [8]. The enterocyte expression was defined as the maximal expression across all crypt-villus zones, based on Moor et al. [11]. Differential gene expression among the sets was performed using Wilcoxon ranksum tests using Benjamini-Hochberg multiple hypotheses correction. FAE up/down-regulated genes were defined as genes with mean expression above 10^{-5}, 2-fold or higher/lower expression respectively in FAE cells compared to villus cells and q-value below 0.1."

The characterisation of the immune cells present in the ILF compartments should be more detailed.

Which T cell types are present?

Where are the dendritic cells and the LTis?

For example, the data on figure 1 suggest that regulatory T cells are enriched on ILFB has Tregs associated genes Foxp3 and Ctla4 are enriched in this area.

We thank the reviewer for these suggestions; we have now explored the spatial distribution of these additional immune cell types using both new computational deconvolution analyses as well as new smFISH experiments. For the computational deconvolution we have parsed a single cell dataset of the small intestine and used the extracted cell-specific transcriptomic signatures as input to the CIBERSORTx deconvolution software (new Figure 1E and new Supplementary Dataset 1). This provided important insights into the spatial distribution of distinct immune subsets in the ILFs:

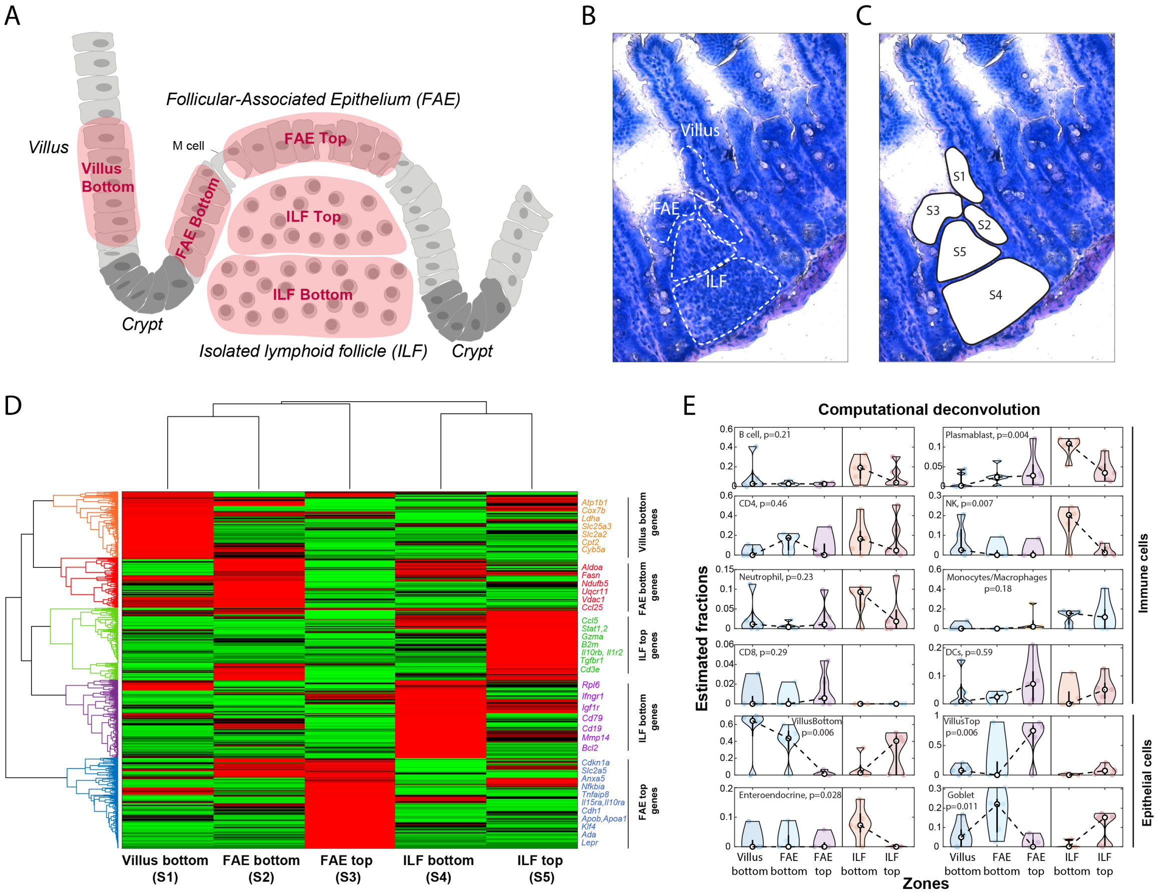

Fig 1. LCM RNA-seq of follicle-associated epithelium (FAE) and isolated lymphoid follicle (ILF). (**A**) An illustration of small intestinal region including FAE, ILF, adjacent villus and crypts, showing the five dissected segments in red: Villus bottom (S1), FAE

bottom (S2), FAE top (S3), ILF bottom (S4) and ILF top (S5). (**B-C**) A bright field microscopy image (20x magnification) of FAE, ILF and the adjacent villus before and after laser dissection of the five segments (S1-S5). (**D**) Clustergram of LCM RNA-seq data showing gene mean expression Z-score of the five segments (S1-S5). Selected genes with high expression are shown on the right side of the clustergram for each cluster, colored according to the cluster color. (**E**) Estimated fractions of distinct cell types, based on computational deconvolution of the LCMseq. White dots are medians, black boxes delineate the 25-75 percentiles. The data used to generate this figure can be found in supporting information S1 and S2 data. P-values computed using Kruskal Wallis tests.

This new analysis is described on page 3:

"To assess the cellular composition of each of segment we performed computational deconvolution using the CIBERSORTx tool [17], that included epithelial and immune single cell-based signatures ([8,11], Fig 1E, S1 data). Among the immune cells, Plasmablasts, B cells, neutrophils and NK cells were more abundant at the ILF bottom, whereas CD8 T cells and dendritic cells were more highly represented (yet not statistically significantly) at the ILF top or the FAE top."

And in the Methods section on page 16:

"Computational deconvolution was performed using CIBERSORTx [17]. CIBERSORTx was run with the "Impute Cell Fractions" analysis module using custom analysis mode with default settings. Mixture file included the LCMseq samples, signature files were extracted based on single cell atlases of the mouse intestine (S1 data). Villus bottom and top enterocytes were defined as the averages of the lower or upper three villus zones in Moor et al. [11] respectively. Goblet cells and enteroendocrine cells signatures were extracted based on Haber et al. [8]. Immune cell signatures were extracted based on Biton et al. [34]. All dendritic cell subtypes were averaged and coarse-grained into one group, as were monocytes and macrophages. Paneth cells, crypt cells and tuft cells were not included in the input signatures due to their expected lower abundance in the tissue compartments analyzed. Both mixture and signature tables were re-normalized to the sum of the

overlapping genes, genes for which the maximal expression among the mixture samples was either 10-fold higher or 10-fold lower than the maximal expression among signature samples were removed and data was subsequently re-normalized so the that each sample sums up to 10^6."

We have also specifically explored the expression of key T-cell genes – Gzma, Cd4, Cd8a and Ctla4. These genes exhibited interesting zonated trends and are shown in the new Figs S2, S3:

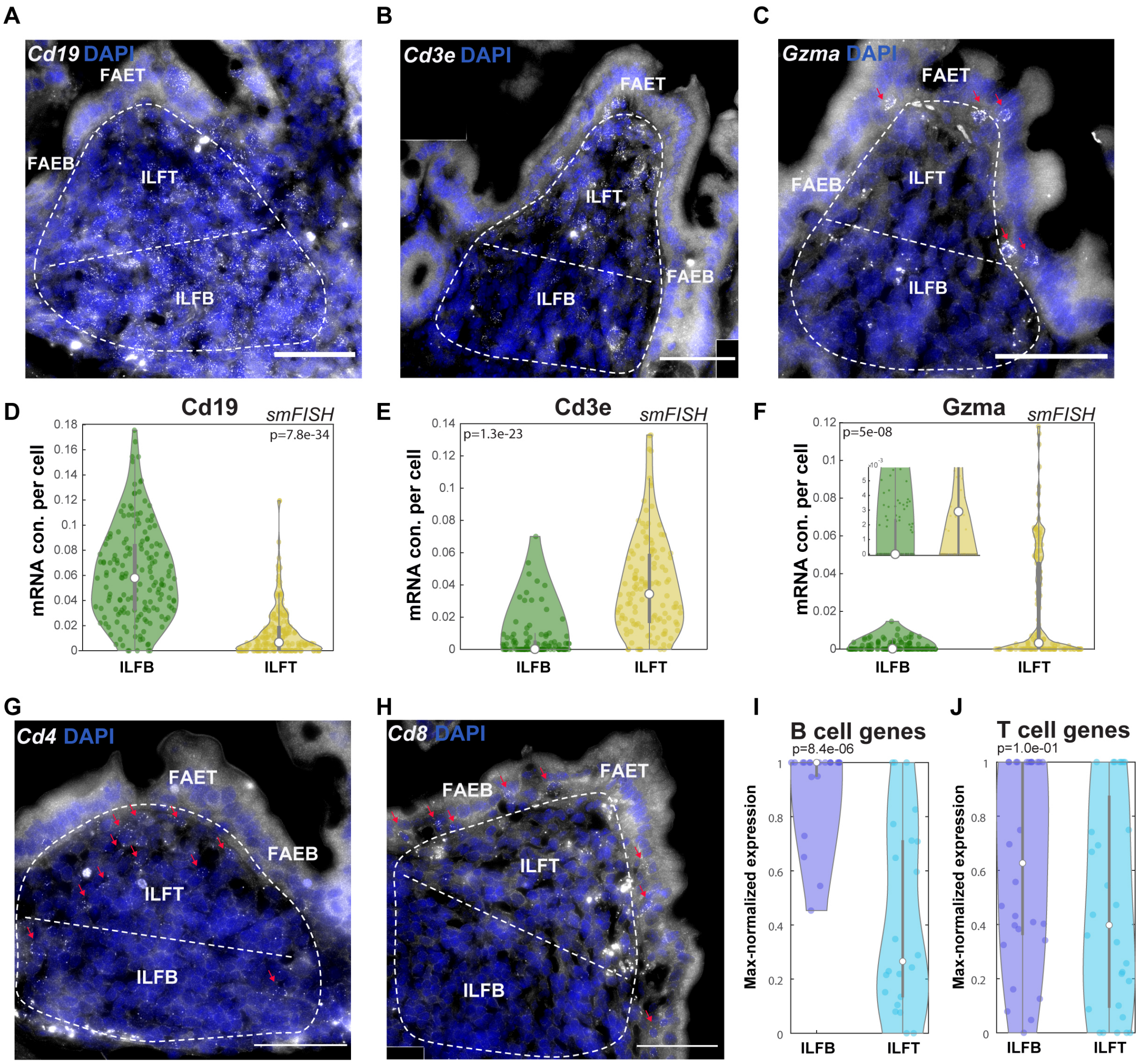

S2 Fig. Zonation of B and T cells in ILF. Related to Fig 1. (**A-C, G-H**) smFISH validations showing increased expression of *Cd19* (**A**) at the ILF bottom and *Cd3e* (**B**), *Gzma* (**C**), *Cd4* (**G**), *Cd8* (**H**) at the ILF top. White dashed lines delimit segment ILF areas and a border line in the middle separates ILFT and ILFB. Red arrows highlight cells with

elevated expression of the respective genes. DAPI staining for cell nucleus in blue. Scale bar- 50 µm. (**D-F**) Violin plots of dot quantifications of smFISH signals of *Cd19*, *Cd3e* and *Gzma*, showing the concentration (con.) of dots (mRNA molecules) per cell area (3-5 individual ILF per mouse for 4 mice). Blow up in **(F)** highlights the majority of the cells with lower expression levels, demonstrating the increase in median *Gzma* levels at the ILFT. (**I**) Violin plots showing max-normalization of B and T cell signature gene expression (Methods). B cell markers are significantly zonated to the ILFB, whereas T cell markers do not exhibit significant bias to the ILFB. White dots are median values, gray boxes delineate the 25-75 percentiles, p-values computed using Kruskal Wallis tests. The data used to generate this figure can be found in supporting information S2, S5 and S6 data.

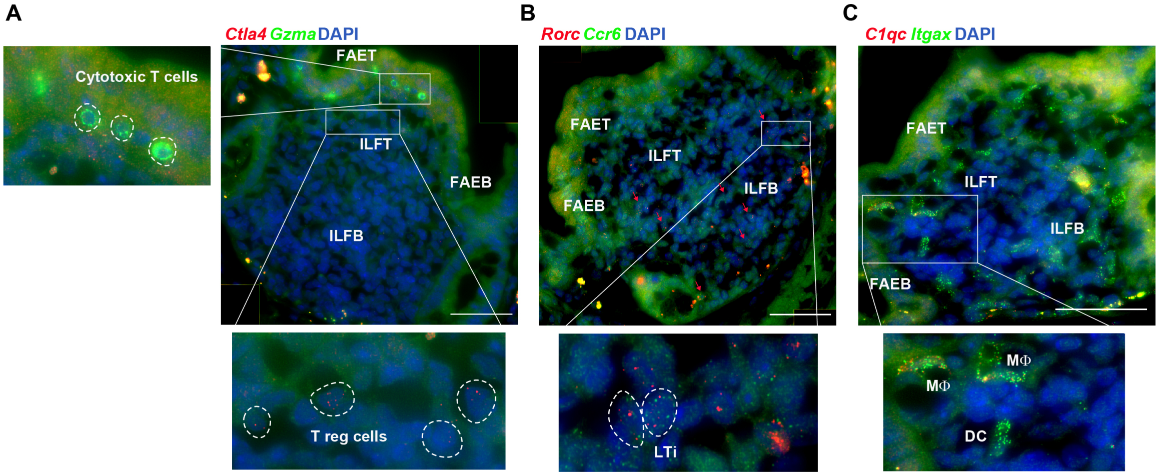

S3 Fig. Immune cell subsets in ILFs. Related to Fig 1. (**A-C**) smFISH images and blow ups showing *Gzma* expressing cytotoxic T cells at the ILF top, infiltrating to the FAET (**A**), as well as *Ctla4*+ regulatory T cells (Treg, **A**) and *Rorc*+*Ccr6*+ LTi cells (**B**) that are scattered throughout the ILFs. Red arrows highlight representative cells. (**C**) Dendritic cells (Itgax+C1qc- cells) and macrophages (Mϕ, Itgax+C1qc+ cells) are radially zonated towards the periphery of the ILF. Scale bar- 50 µm.

These are discussed on page 3:

"Consistently, we identified higher expression of different B-cell markers, such as *Cd19*, at the bottom of the ILF (S2A,D,I Fig). In contrast, T cell markers did not show a spatial

bias towards a specific zone in the ILF (S2J Fig). Rather, using smFISH we found that *Cd3e, Cd4 and Cd8,* classic T cell markers, as well as *Gzma*, a marker of cytotoxic CD8+ T cells were highly abundant at the ILF top. (S2B,C,E-H Fig). Consistently, CD8+ and Gzma+ cells were abundantly intercalated across the FAE top (S3A Fig), whereas CD4+ cells (S2G Fig) as well as the Treg marker Ctla4 were zonated towards the ILF top (S3A Fig). The spatial pattern of lymphocytes in the ILF resembles the architecture previously observed in Peyer's patches, which exhibit a core of B cells and a mantle of T cells [18]."

To identify LTis, we have now performed co-smFISH using the markers Rorc and Ccr6. We identify these cells as rare and scattered, without specific apparent zonation patterns. To characterize dendritic cells and macrophages, we have also now performed smFISH experiments with probe libraries for the genes Itgax and C1qc. The combined visualization of both of these genes enables identification of dendritic cells (Itgax+C1qc- cells) and macrophages (Itgax+C1qc+ cells). Interestingly, we found that both dendritic cells and macrophages have a radial zonation, enriched towards the periphery of the ILFs. These results are shown in the new Figure S3B,C above and described on page 3:

"Using smFISH, we further identified scatter localization of Rorc+Ccr6+ Lymphoid Tissue induced cells (LTi cells [19], S3B Fig), as well as radial zonation of dendritic cells (identified as Itgax+C1qc- cells) and macrophages (Itgax+C1qc+ cells) [20] towards the periphery of the ILF (S3C Fig)"

On this subject, on Fig Sup 3E, CD3a is increased at ILFT (not at ILFB) - the figure legend is incorrect while main text is correct. In addition, on Fig Sup 3E, according to the violin plot, the medians for Gmza for ILFT and ILFB seem very similar.

We thank the reviewer for noting the typo regarding Cd3e, which we have now corrected. We have also included a blow-up of the y axis highlighting the elevated median levels of Gzma in ILFT compared to ILFB (Figure S2F pasted above).

Using their RNAseq data and SmFISH authors identified Lepr+ telocytes. What's the function of these telocytes?

As the authors mention it would be interesting to characterise IFLs in disease models and germ free animals. In addition, those this compartmentalisation also occurs in human ILFs?

These are fascinating future directions which we describe in the Discussion on page 12:

“What are the factors that could elicit the distinct gene expression programs at the FAE top? FAE enterocytes are localized at around the same physical distance from the crypt as villus bottom enterocytes. Moreover, previous work has shown that FAE eneterocytes are continuously migrating and shedding from the FAE tip, similarly to villus enterocytes [30]. Yet, the FAE operates under a unique microenvironment compared to the villus bottom. Luminal bacterial concentrations at the FAE tip should be higher due to the thinner mucous layer and reduced secretion of anti-microbial peptides, and therefore more similar to the luminal microenvironment at the villus tip [11]. We found that, unlike villus-tip enterocytes, FAE top enterocytes are in contact with *Lepr*+ telocytes, which could provide different niche signals than their villus tip *Lgr5*+ telocytes counterparts. Indeed, the expression of the purine-metabolism immune-modulatory genes *Ada*, *Nt5e* and *Slc28a2* at the villus tip seems to be controlled by *Lgr5*+ telocytes [28], potentially explaining their reduced expression at the FAE top. Our study forms the basis for the future exploration of the regulatory molecules that shape FAE zonation. It will be interesting to expand our study to cryptopatches and colonic ILFs, as well as to the characterization of ILFs in perturbed states such as germ-free mice and in models of inflammatory diseases.”

The manuscript describes the bioinformatic analysis of the data generated - I don't have the expertise to comment on that.

Rev. 2:

In this study, the author use Laser-capture dissection and RNA-sequencing of a few initial samples to identify expression programs in different areas of isolated lymphoid follicles (ILF) in the mouse epithelium. They use the genes identified in this approach to

characterize spatial expression using extensive single molecule FISH. As this small structure has not been investigated on a molecular level with respect to its cell type compositions, the author's analyses are certainly helpful to better understand it and its relevance for immune surveillance in the gut. However, I find the manuscript and the data not advanced enough to be really helpful for a larger readership at this point.

1) I think technically the LCM-seq is fine, but the experimental design is unclear and the number of samples is too low. The number of replicates is not even mentioned in the main text, the figure is a very ad hoc description of the central aspect of the manuscript and statistical analyses that take into account biological replicates and the repeated measures is also totally missing.

We thank the reviewer for this comment that prompted us to significantly expand our study. We have now performed all smFISH validation experiments on two additional mice, yielding four mice in total. These are now described in each figure caption. We have also clarified the numbers of replicates for the LCM experiments on page 13-14:

"In total, twenty five LCM samples were collected for RNA sequencing, containing five repeats from two mice (rep1-rep2 for mouse#1 and rep3-rep5 from mouse#2). Each repeat included a set of five areas: FAE top, FAE bottom, Villus bottom, ILF top and ILF bottom."

It is important to note that LCM is a very noisy measurement technique that renders it very hard to extract non-parametric (e.g. Kruskal Wallis) p-values for individual genes. Nevertheless, meta-analyses on this data provides clear statistically significant patterns, e.g. the new computational deconvolution analysis suggested by the reviewer, see below, the statistical enrichment of processes for distinct clusters (Supplementary Dataset 3), and the spatial trends for groups of genes such as B-cell genes, M-cell genes, ribosomal proteins or solute carriers (S2I Fig, S4A Fig, S6 Fig respectively). The comprehensive smFISH analyses of the trends suggested by the LCM results of either such groups or individual genes in the LCM, now performed on four mice, clearly validate and substantiate the main findings, as we write on page 3:

2"We used these LCMseq expression programs as a basis for extensive smFISH validations, to establish their statistical power."

We have now clearly provided statements regarding the significance analyses of the LCM results, namely when p>0.05 in each of the displayed panel captions.

2) The structure of how these initial results are followed-up with smFISH is very unclear, at least to me as a non-specialist in intestine cell types. Some genes are picked rather ad hoc from the list, subjected to smFISH and then interpreted. I can't detect a transparent structure in the manuscript which questions are addressed and how they are answered. A potentially more transparent way to tackle this would be to deconvolute the LCM-seq data to predict certain cell-types and then confirm them with smFISH… but this is certainly not the only possibility.

We thank the reviewer for this comment, which prompted us to perform computational deconvolution on our data, which we believe added important insights and presents a more unbiased way to approach the data. To this end, we have parsed a single cell dataset of the small intestine and used the extracted cell-specific transcriptomic signatures as input to the CIBERSORTx deconvolution software (new Figure 1E and new Supplementary Dataset 1). This provided important insights into the spatial distribution of distinct immune subsets in the ILFs:

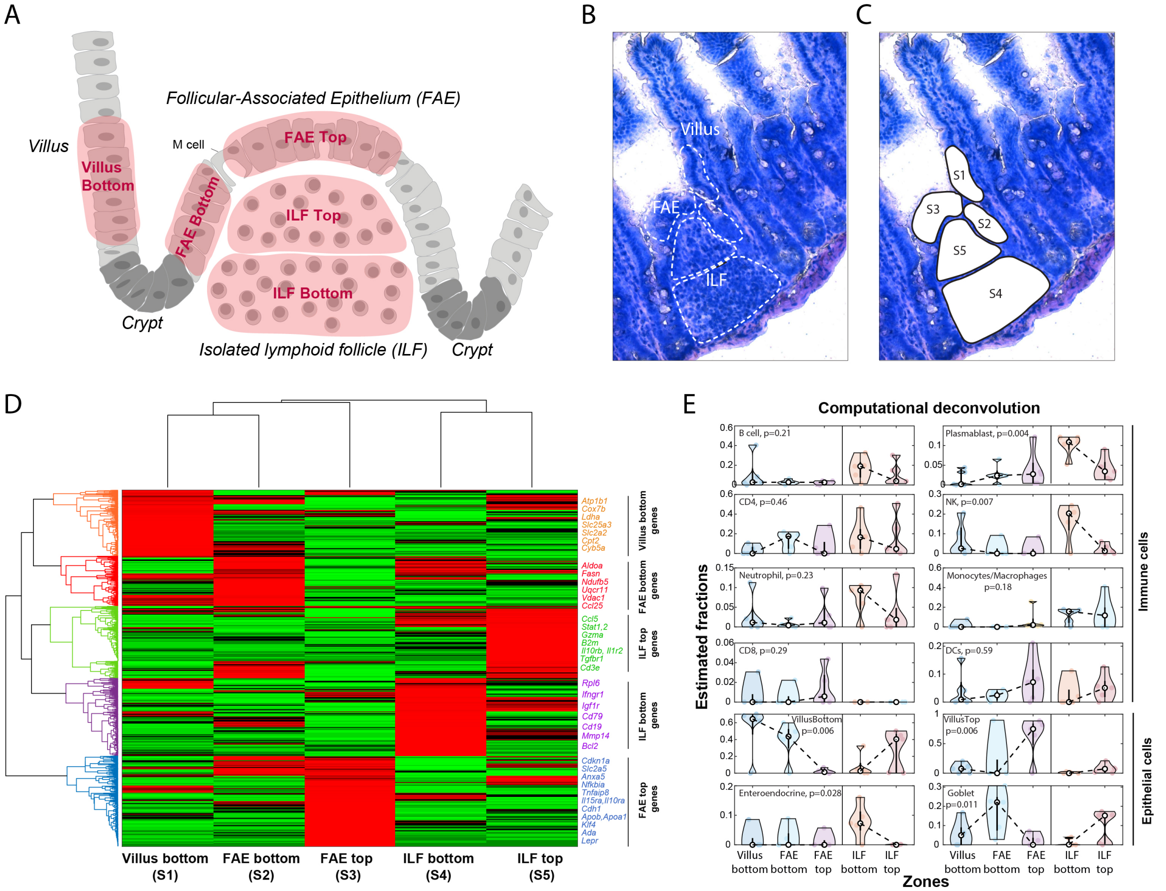

Fig 1. **LCM RNA-seq of follicle-associated epithelium (FAE) and isolated lymphoid follicle (ILF)**. (**A**) An illustration of small intestinal region including FAE, ILF, adjacent villus and crypts, showing the five dissected segments in red: Villus bottom (S1), FAE bottom (S2), FAE top (S3), ILF bottom (S4) and ILF top (S5). (**B-C**) A bright field microscopy image (20x magnification) of FAE, ILF and the adjacent villus before and after laser dissection of the five segments (S1-S5). (**D**) Clustergram of LCM RNA-seq data showing gene mean expression Z-score of the five segments (S1-S5). Selected genes with high expression are shown on the right side of the clustergram for each cluster, colored according to the cluster color. (**E**) Estimated fractions of distinct cell types, based on computational deconvolution of the LCMseq. White dots are medians, black boxes delineate the 25-75 percentiles. The data used to generate this figure can be found in supporting information S1 and S2 data. P-values computed using Kruskal Wallis tests.

This new analysis is described on page 3:

"To assess the cellular composition of each of segment we performed computational deconvolution using the CIBERSORTx tool [17], that included epithelial and immune single cell-based signatures ([8,11], Fig 1E, S1 data). Among the immune cells, Plasmablasts, B cells, neutrophils and NK cells were more abundant at the ILF bottom, whereas CD8 T cells and dendritic cells were more highly represented (yet not statistically significantly) at the ILF top or the FAE top."

And in the Methods section on page 16:

"Computational deconvolution was performed using CIBERSORTx [17]. CIBERSORTx was run with the "Impute Cell Fractions" analysis module using custom analysis mode with default settings. Mixture file included the LCMseq samples, signature files were extracted based on single cell atlases of the mouse intestine (S1 data). Villus bottom and top enterocytes were defined as the averages of the lower or upper three villus zones in Moor et al. [11] respectively. Goblet cells and enteroendocrine cells signatures were extracted based on Haber et al. [8]. Immune cell signatures were extracted based on Biton et al. [34]. All dendritic cell subtypes were averaged and coarse-grained into one group, as were monocytes and macrophages. Paneth cells, crypt cells and tuft cells were not included in the input signatures due to their expected lower abundance in the tissue compartments analyzed. Both mixture and signature tables were re-normalized to the sum of the overlapping genes, genes for which the maximal expression among the mixture samples was either 10-fold higher or 10-fold lower than the maximal expression among signature samples were removed and data was subsequently re-normalized so the that each sample sums up to 10^6"

3) If the paper should be accessible to a wider readership beyond the immediate field the jargon and abbreviations need to be reduced and better introduced.

We have now modified the text to improve clarity, specifically avoiding jargon related to the transcriptomics part.
