# Supplementary material for: Spatial gene expression maps of the intestinal lymphoid follicle and associated epithelium identify zonated expression programs
Source: PLoS Biol. 2021 Oct 11;19(10):e3001214. doi: 10.1371/journal.pbio.3001214 (PMC8530339; doi:10.1371/journal.pbio.3001214)
Supplement: Attachment — Submitted filename: response_to_comments_september_2021.pdf [file pbio.3001214.s014.pdf]

Dear Ines

We thank you for this positive assessment of our revision.
We have now made all of the final correction needed. The changes have been underlined in the manuscript text and are described below:

1) We have changed the title of the paper as suggested.
2) We have made the GEO publicly available.
3) We have added a reference that clarifies the use of Itgax/C1qc for the resolution of macrophages and dendritic cells (new reference 21) as requested by the reviewer.

Sincerely

Shalev Itzkovitz
